# Association between Human Body Composition and Periodontal Disease

**DOI:** 10.5402/2011/863847

**Published:** 2011-11-02

**Authors:** Yagoub Salekzamani, Adileh Shirmohammadi, Mohammad Rahbar, Seyed-kazem Shakouri, Farough Nayebi

**Affiliations:** ^1^Physical Medicine and Rehabilitation Research Center, Tabriz University of Medical Sciences, Golgasht Street, Tabriz 51666-14691, Iran; ^2^Department of Periodontics, Dental Faculty, Tabriz University of Medical Sciences, Tabriz 51666-14711, Iran

## Abstract

Obesity in humans might increase the risk of periodontitis. The aim of the present study was to examine the relationship between body composition of males and their periodontal status. AS total of 150 males (aged 30–60) were selected: 31 were periodontally healthy, 45 had gingivitis, 39 had initial periodontitis, and 35 suffered from established periodontitis. BMI (body mass index), WC (waist circumference), and body composition parameters (consisting of body water, body fat, and skeletal muscle and bone mass) were measured. After adjusting for age, history of diabetes, smoking, physical activity status, and socioeconomic status, statistically significant correlations were found between periodontitis and BMI, WC, and body composition. There was only a statistically significant difference between the periodontal health and established periodontitis; that is, periodontal disease in mild forms (gingivitis) and initial periodontitis do not influence these variables (BMI, WC, and body composition parameters) and only the severe form of the disease influences the variables. These data suggest that there is a considerable association between severe forms of periodontal disease in males and their body composition, but this preliminary finding needs to be confirmed in more extensive studies.

## 1. Introduction

Obesity has been associated with many serious, life-threatening medical conditions, including cardiovascular disease [1], various cancers [2], and diabetes [3] as well as endocrine [4] and musculoskeletal diseases [5]. Central obesity is a risk factor for metabolic syndrome, a group of conditions or risk factors that increase a subject's risk for cardiovascular disease [6].

Being overweight or obese has also been associated with an increased risk for periodontal disease [7]. Periodontal disease is a common infectious disease associated with gram-negative anaerobic bacteria, characterized by inflammation and destruction of periodontal tissues [8].

Studies have described an association between periodontal disease and obesity [7], increased body mass index (BMI) [9], and increased waist circumference [10]. In addition, Shimazaki et al. [11] reported that metabolic syndrome increases the risk for periodontitis, while increased levels of serum resistin, an adipokine secreted from adipose tissues, was significantly associated with periodontitis in a population of Japanese women [12].

Although a large number of studies have evaluated the effect of periodontal disease on obesity, no studies have, so far, focused on the association between the severity of this chronic disease and body composition. Therefore, the present study was undertaken to determine the influence of body composition on periodontal disease in males.

The main objective of the present study was to evaluate a hypothesis: body composition can have a detrimental effect on severity of periodontal disease.

In case of an association between these two, future studies can evaluate this hypothesis.

## 2. Methods

### 2.1. Design

The present study was an analytical cross-sectional study.

### 2.2. Study Population

The study population consisted of men aged 30 to 60, who had referred to the Faculty of Dentistry, Tabriz University of Medical Sciences from April to November 2010. Exclusion criteria included systemic conditions such as diabetes, cardiovascular disorders, or environmental factors such as tobacco use, poor or severe physical activity and men who had received periodontal treatment during the previous 3 months.

A total of 150 men met the criteria for participation in the study, and a census procedure was carried out in order to acquire and record data from the subjects during the period of the study. The study design was approved by the Ethics Committee and supported by the Research Deputy of Tabriz Medical Faculty. The nature of this investigation was explained to the patients in detail, and the patients signed an informed consent form.

### 2.3. Data Collection

Clinical histories were taken by one of the authors to ensure that none of the aforementioned exclusion criteria was present. Periodontal measurements were taken by AS classification on the basis of gingival and plaque indices (Ainamo and Bay [13]) and means of attachment loss. In this index, bleeding from the gingival margin and visible plaque has a score of “1,” while absence of bleeding and no visible plaque have a score of “0.”

Attachment loss (the distance from the CEJ to the base of pocket) was measured with a Williams periodontal probe (PWD, Hu-Friedy Immunity, USA) for all the existing teeth on four surfaces (buccal, mesial, distal, and palatal or lingual), and the means were calculated. Based on the results, the subjects were divided into 4 groups as follows.

Group 1: normal with no gingival inflammation (gingival plaque index, GPI = 0) and no attachment loss.
Group 2: simple gingivitis with gingival inflammation (GPI = 1) and no attachment loss.
Group 3: initial periodontitis with gingival inflammation (GPI = 1) and attachment loss of <2 mm.
Group 4: established periodontitis with gingival inflammation (GPI = 1) and attachment loss of >2 mm.

Weight was measured in kilograms (kg), height in centimeters (cm), and BMI in kg/m^2^. Waist circumference measurements were taken at the level of the umbilicus in centimeters (cm).

Fat mass and skeletal muscle mass were determined from the impedance and conductance measures of the bioelectrical impedance analysis (BIA). Because fat-free mass is composed of water, proteins, and electrolytes, conductivity is greater in fat-free mass than in fat mass [14]. Resistance and reactance are used to estimate body water, fat and skeletal muscle, and bone mass. These parameters were measured with Diagnostic Scale-Beurer BG 56.

### 2.4. Statistical Analysis

Means of these variables were calculated for each group and compared using one-way ANOVA. If there were statistically significant differences between the groups, post hoc tests were used for further analysis. A *P* value of <0.05 was considered significant.

## 3. Results

A total of 150 men (aged 30–60) participated in the present study, of which 31 were periodontally healthy, 45 had gingivitis, 39 had initial periodontitis, and 35 suffered from established periodontitis. ANOVA indicated no significant differences in the age of the subjects between the four groups (*P* > 0.05).

Statistical analysis using one-way ANOVA demonstrated statistically significant associations between BMI, waist circumference, body water, fat mass, skeletal muscle mass and bone mass, and the periodontal status of patients from normal periodontium and gingivitis, to initial periodontitis, and to established periodontitis.  Table 1 summarizes the values relating to BMI, waist circumference, body water, body fat, and skeletal muscle and bone mass in relation to patients' periodontal status.

A post hoc Tukey test revealed that there was only a statistically significant difference between periodontal health and established periodontitis; that is, periodontal disease in mild forms (gingivitis) and initial periodontitis do not influence these variables and only the severe form of the disease influences the variables (Table 2).

## 4. Discussion

This study was designed to determine the relationship between periodontal disease of males and their BMI, WC, and body composition after adjusting for age, history of diabetes, smoking, physical activity, and socioeconomic status. 

In the periodontitis groups (initial and established forms), the average BMI was significantly greater than the healthy and gingivitis groups, which is consistent with the results of a systematic review by Chaffee and Weston [15]. His study suggested a greater mean clinical attachment loss among obese individuals, a higher mean body mass index (BMI) among periodontal patients, and a trend of increasing odds of prevalent periodontal disease with increasing BMI. However, Kim et al. [16] showed no association between BMI and periodontitis. Obese people with BMI ≥25 had an adjusted odds ratio (OR) of 0.991 (0.806 to 1.220) for having periodontitis. This discrepancy might be attributed to the age of the participants. Participants in Kim et al. study were younger than those in ours.

Our study showed higher waist circumferences in periodontitis patients compared to healthy and gingivitis groups, which is consistent with the results reported by Khader et al. [17], who suggested that periodontitis was more prevalent among subjects with high waist circumference.

According to another study, there was a significant correlation between both BMI and WC and CAL, GI, and CPI in females. In males, a significant correlation was only recorded between WC and GI and CPI. Overall and abdominal obesities in young adult females and abdominal obesity in males were significantly associated with periodontal disease [18]. 

What distinguishes the present study from other studies on the subject is the evaluation of body composition parameters in men suffering from varying degrees of periodontal disease.

The percentage of body fat was least in the healthy group and most in the established periodontitis groups (21.1 and 26.8, resp.). A Japanese study found that the risk of periodontitis for each 5% increase in body fat was 1.3 (*P* = 0.02) after adjusting for age, gender, oral hygiene status, and smoking history [19]. One limitation of our study was that we could not measure body fat distribution. Body fat distribution—‘‘where fat?” in addition to ‘‘how fat?”—is likely to be a critical epidemiological factor in diseases of the oral cavity. Wood showed that upper body fat localization is a significant risk factor for periodontal disease and suggested that fat metabolism may play a key role in these relationships [20]. This association between periodontitis and the role of fat should be investigated in more detail in future studies.

The percentage of body water decreased as the severity of periodontal disease increased. In this context, it should be pointed out that these are the first data obtained for the evaluation of association between body water and periodontal disease. Therefore, a comparison with other studies is not possible.

In the present study, skeletal muscle percentage was significantly higher in the healthy and gingivitis groups compared to the initial and established periodontitis groups (43.4, 43.0, 38.7, and 39.5, resp.). It is difficult to explain this finding, but it might be related to protein deficiency. Muscles are made primarily from proteins. Protein deprivation has been shown to cause changes in the periodontium of experimental animals [21]. The following observations have been made in protein-deprived animals: degeneration of the connective tissue of the gingiva and periodontal ligament, osteoporosis of alveolar hone, impaired deposition of cementum, delayed wound healing, and atrophy of the tongue epithelium. Similar changes occur in the periosteum and bone in other nonoral areas. Osteoporosis results from reduced deposition of osteoid, depletion of osteoblasts, and impairment in the morphodifferentiation of connective tissue cells to form osteoblasts, rather than from increased osteoclastic activity. Protein deficiency also intensifies the destructive effects of bacterial plaque and occlusal trauma on periodontal tissues, but the initiation of gingival inflammation and its severity depend on bacterial plaque [22]. In other words, protein deprivation results in periodontal tissues that lack integrity and, as a result, are more vulnerable to breakdown when challenged by bacteria. 

The present study indicated that periodontal disease is associated with bone mass. Subjects in the healthy periodontium group had significantly more bone mass compared to those in the gingivitis and periodontitis groups. Osteoporosis and periodontitis are diseases which affect a large number of females and males, with the incidence increasing with advancing age. Osteopenia is a decrease in bone mass due to an imbalance between bone resorption and formation, favoring resorption, resulting in demineralization and osteoporosis. Osteoporosis is a disease characterized by low bone mass and fragility and a consequent increase in fracture risk. Periodontitis is characterized by inflammation of the supporting tissues of the teeth, resulting in resorption of the alveolar bone as well as loss of the soft tissue attachment to the tooth and is a major cause of tooth loss and edentulism in adults [23]. The relationship of osteopenia to oral bone loss and periodontal disease has been addressed in some studies. A study by Yoshihara et al. [24] suggested that there is a significant relationship between periodontal disease and general bone mineral density. Better understanding of this relationship may aid health care providers in their efforts to detect and prevent osteoporosis and periodontal disease. Increased dialogue among medical and dental professionals is increasingly important in achieving and maintaining patients' optimal health.

It should be pointed out that the present study was carried out to establish an association between body composition and periodontal disease and it cannot be considered a causative agent.

## 5. Conclusion

The present study suggests that there is a considerable association between severe forms of periodontal disease in males and their body composition, but this preliminary finding needs to be confirmed in more extensive studies.

## Figures and Tables

**Table 1:** BMI, waist circumference, and body composition based on patient's periodontal condition (mean ± SD).

	BMI (kg/m^2^)	Waist circumference (cm)	Body water (%)	Body fat (%)	Skeletal muscle (%)	Bone mass (kg)
Normal (*N* = 31)	25.0 ± 2.0	88.1 ± 1.2	57.1 ± 1.1	21.1 ± 2.0	43.4 ± 3.9	11.1 ± 1.0
Simple gingivitis (*N* = 45)	24.5 ± 2.9	86.4 ± 5.8	57.0 ± 4.0	20.3 ± 5.9	43.0 ± 4.9	10.3 ± 1.1
Initial periodontitis (*N* = 39)	27.3 ± 2.1	96.3 ± 5.1	53.5 ± 3.4	25.8 ± 4.5	38.7 ± 0.4	9.8 ± 0.7
Established periodontitis (*N* = 35)	28.3 ± 1.4	96.2 ± 4.5	52.6 ± 1.6	26.8 ± 1.2	39.5 ± 0.3	10.1 ± 1.3
*P* (ANOVA)	0.000*	0.000*	0.000*	0.000*	0.000*	0.000*

*Indicates statistical significance based on *P* < 0.05.

BMI: body mass index (kg/m^2^).

**Table 2:** Comparison between normal periodontal status and various stages of periodontal disease, related variables (data are expressed in *P* value).

Periodontal status	Variables					
	BMI	Waist circumference	Body water	Body fat	Skeletal muscle	Bone mass
Normal periodontium						
Gingivitis	0.841	0.402	0.998	0.855	1.000	0.008*
Initial periodontitis	0.000*	0.000*	0.000*	0.000*	0.000*	0.000*
Established periodontitis	0.000*	0.000*	0.000*	0.000*	0.000*	0.003*
Gingivitis						
Initial periodontitis	0.000*	0.000*	0.000*	0.000*	0.000*	0.162
Established periodontitis	0.000*	0.000*	0.000*	0.000*	0.000*	0.955
Initial periodontitis						
Established periodontitis	0.272	0.999	0.556	0.723	0.682	0.464

*Indicates statistical significance based on *P* < 0.05.
